# Reversal of Early Diabetic Nephropathy by Islet Transplantation under the Kidney Capsule in a Rat Model

**DOI:** 10.1155/2016/4157313

**Published:** 2016-09-20

**Authors:** Yunqiang He, Ziqiang Xu, Mingshi Zhou, Minmin Wu, Xuehai Chen, Silu Wang, Kaiyan Qiu, Yong Cai, Hongxing Fu, Bicheng Chen, Mengtao Zhou

**Affiliations:** ^1^Key Laboratory of Surgery, The First Affiliated Hospital of Wenzhou Medical University, Wenzhou 325000, China; ^2^Department of Transplantation, The First Affiliated Hospital of Wenzhou Medical University, Wenzhou, Zhejiang Province 325000, China; ^3^School of Pharmacy, Wenzhou Medical University, Wenzhou, Zhejiang Province 325000, China; ^4^Department of Surgery, The First Affiliated Hospital of Wenzhou Medical University, Wenzhou 325000, China

## Abstract

*Objective*. Diabetic nephropathy (DN) is a common microvascular complication of diabetes mellitus, and insulin therapy has many side effects in the treatment of DN. Islet transplantation has emerged as a promising therapy for diabetic patients. This study was established to investigate its advantageous effects in a rat model of early DN.* Methods*. Streptozotocin was administered to the rats to induce diabetes. Twelve weeks later, the diabetic rats were divided into 3 groups: the islet-transplanted group (IT group), the insulin-treated group (IN group), and the untreated group (DN group). Renal injury and kidney structure were assessed by urinalysis and transmission electron microscopy (TEM) detection. Immunohistochemical staining and western blotting were performed to assess renal fibrosis levels.* Results*. The early DN features were reversed and the glomerular filtration barrier and basement membrane structures were improved at 4 weeks after islet transplantation. The urine microalbumin-to-creatinine ratio (ACR), protein-to-creatinine ratio, and mean thickness of the glomerular basement membrane (GBM) were significantly decreased in the IT group. The expression of renal fibrotic factors was also significantly decreased.* Conclusions*. These data suggest that early DN can be reversed after islet transplantation, and they may facilitate the development of a clinical therapeutic strategy for human diabetes mellitus.

## 1. Introduction

Diabetic nephropathy (DN) is one of the common microvascular complications of diabetes, and it is characterized by expansion of the mesangial matrix, thickening of the glomerular basement membrane (GBM), persistent proteinuria, and progressive renal dysfunction in diabetic patients with hyperglycemia [1]; it has been estimated that approximately 20%–30% of type 1 and type 2 diabetic patients develop DN [2]. It is considered the leading cause of end-stage renal disease (ESRD) in many developed countries, and, in China, it accounts for more than 25% of all cases of ESRD [3]. The occurrence and development of DN seriously affect the quality of life, prognosis, and even survival of diabetic patients [4, 5].

Components of the high glucose milieu in diabetes are the main causes of renal injury in DN; therefore, controlling the plasma glucose level is considered the most crucial goal during treatment. In addition to patients with type 1 diabetes mellitus, those with type 2 diabetes might also eventually require insulin therapy [6]. An increasing number of studies have found that although diabetic patients start taking medication or insulin at the early disease stage, most of them still develop progressive DN, indicating that appropriate therapeutic targets and additional treatments are urgently needed [7]. In the past few decades, with a general understanding of diabetes mellitus, various therapeutic protocols have been proposed to treat diabetes mellitus or its late complications, including vascularized, whole-pancreas transplantation and islet cell transplantation [8]. Recent studies have suggested that pancreas or islet transplantation can ameliorate the clinical signs of diabetes mellitus, stabilize further progression, and even reverse the related renal injury in early DN [9–11], primarily because it is more suitable for use under physiological conditions* in vivo*. Nevertheless, the complexity of the vascularized whole-pancreas transplantation procedure may, along with the preexisting diseases present in many diabetic patients, increase the risks of operative and postoperative complications [7].

Recently, with gradual advances in methods of islet isolation, islet cell transplantation has progressed from the laboratory to the clinical setting [12]. Compared with whole-pancreas transplantation, it is a simpler operation with lower morbidity, and it has become an attractive alternative therapeutic method to conventional insulin injection or pancreas transplantation for treating diabetes and its complications [13]. The purpose of this study was to investigate the efficacy of islet transplantation for reversing early DN and to establish whether this treatment is superior to insulin therapy for the regression of DN. The differences between islet transplantation and pancreas transplantation are also discussed in our study.

Furthermore, commonly measured parameters were assessed, as described in previous studies, including the blood glucose level, body weight, and the urine protein level. Microscopic pathological changes of the glomerulus and podocytes during treatment were also examined. Podocyte abnormalities are crucial components of progressive renal injury and are known as good predictors of early DN [12]. Studies of the role of podocytes in renal injury have highlighted their importance in recent years. In this study, we have elaborated upon the significance of the recovery of podocyte structure and function during the treatment of diabetic nephropathy. Additionally, the roles of profibrogenic factors and interstitial cytokines, including TGF-*β*1, HGF, CTGF, *α*-SMA, and MCP-1, during the progression of renal fibrosis were assessed in early DN. The links between these fibrotic factors and the possible mechanisms of podocyte injury in early DN are also discussed in this study.

## 2. Materials and Methods

### 2.1. Animal Model and Groups

A total of 50 male Sprague-Dawley (SD) rats weighing 200–220 g were provided by the Experimental Animal Center of Wenzhou Medical University, and some of the rats (*n* = 12) were used as islet donors. All rats were housed with a 12 h light/dark cycle at 24°C ± 1°C and provided with food* ad libitum* for a week before initiation of the study. All animal experiments were approved by the Wenzhou Medical University management Committee for Medical Laboratory Animal Sciences. A rat diabetic model was induced by a single peritoneal injection of streptozotocin (STZ) (55 mg/kg of body weight) in sodium citrate buffer (pH 4.5) after fasting overnight. The plasma glucose concentration was measured in a drop of tail vein blood using an Accu-Chek glucometer (Roche Diagnostics, Indianapolis, IN). One week later, when the nonfasting blood glucose concentration was ≥16.67 mmol/L for 3 days, the experimental diabetic rat model was established [14]. After 12 weeks, the urine ACR and protein-to-creatinine ratio were determined, and transmission electron microscopy (TEM) was performed to assess whether the early DN rat model had been successfully established. Next, the rats were randomly divided into three different groups: in the untreated group (DN group, *n* = 8), the rats were left untreated and studied 4 weeks later; in the islet-transplanted group (IT group, *n* = 8), the rats underwent islet transplantation under the kidney capsule and were studied 4 weeks later; and, in the insulin treatment group (IN group, *n* = 8), the rats were given glargine insulin (Wanbang Pharmaceuticals, Jiangsu, China) by subcutaneous injection at 9 a.m. and 9 p.m. every day for the following 4 weeks (3 U each). The normal control rats were regarded as the control group (NC group, *n* = 8).

### 2.2. Islet Isolation and Purification

Islets were isolated from the rat pancreas using previously described methods [15]. Briefly, the rats were anesthetized by intraperitoneal injection of chloral hydrate, and laparotomy was performed to expose the pancreas. The entrance of the common bile duct into the intestine was located and ligated, and 8 mL collagenase V (0.8 mg/mL, dissolved with Hanks solution) was injected into the common bile duct by retrograde intubation. When the pancreas was fully inflated, it was separated from the surrounding tissues with surgical tweezers, transferred to a 50 mL centrifuge tube, and digested for 10–15 min at 37 ± 0.5°C. After digestion, the tissue was washed with Hanks' solution three times. Then, the islets were purified by density gradient centrifugation (Histopaque -1119 and Histopaque -1077) at 2000 rpm for 5 min. The supernatant was poured into a new centrifuge tube and transferred to a black glass culture dish for manual selection of islets. The final purified islets were cultured in RPMI-1640 (Gibco, Carlsbad, CA, USA) containing 10% fetal bovine serum (FBS; Gibco, Invitrogen, Inc., USA) at 37°C and 5% CO_2_.

### 2.3. Islet Counting, Equivalent Calculation, and Activity Evaluation

The purified islets were adjusted to an appropriate concentration in the culture medium and transferred to a small culture dish with a 2 mm lattice for their quantification under a microscope. Based on the methods of Lembert [16] and others, the cell clusters were counted, and the diameters were measured with a microscope eyepiece scale. Total islet equivalents (IEQ) were calculated according to the appropriate formula [16]. A single aliquot of 100 freshly isolated islets was aspirated into a 200 *μ*L pipette tip and transferred to a small culture dish. The activity of the islets was evaluated by fluorescein diacetate-propidium iodide (FDA-PI) staining under an inverted fluorescence microscope. The activity ratio was determined using 100 islet cell clusters to estimate that of the total final purified islets.

### 2.4. Islet Transplantation under the Kidney Capsule

Based on the method reported by Napoli et al. [17], the final purified islets of approximately 800–1000 IEQ were aspirated into a 1 mL syringe connected to P50 polyethylene tubing, and the islet was transferred to the head end. The recipient rat was anesthetized by intraperitoneal injection of chloral hydrate, the left flank was shaved, and the kidney was exposed through a small lumbar incision. Capsulotomy of the kidney was performed on the caudal outer surface, and the tip of the polyethylene tubing was inserted and advanced gently under the kidney capsule. The surface of the kidney was kept moist with saline during the procedure. The islet in the tubing was pushed out slowly and carefully, and the tube was removed after complete transfer of the islet into the capsule.

### 2.5. Tissue and Urine Sampling

The nonfasting blood glucose levels and body weights of the rats in each group were measured once per week. Individual rat metabolic cages were used to collect random and 24 h urine samples twice per week. The urine protein concentration was measured using the sulfosalicylic acid precipitation method, and the creatinine concentration was determined with a creatinine assay kit (Bioassay Systems, Hayward, CA). Albuminuria was determined using a rat albumin-specific ELISA kit (Exocell Laboratories, Philadelphia, PA). The small pieces of renal cortex obtained from the kidney tissue were cut into small cubes and fixed in 2.5% glutaraldehyde. The remaining tissue was fixed overnight in 4% paraformaldehyde for histopathological staining. After gradient alcohol dehydration and incubation with xylene transparent agent, the kidney tissue was embedded in paraffin and cut into 5 *μ*m sections using a microtome.

### 2.6. Immunohistochemistry Staining

Following the IHC staining protocol described by Pichaiwong et al. [18], the paraffin tissue sections were incubated overnight with primary antibodies to TGF-*β*1, HGF, CTGF, *α*-SMA, MCP-1, and synaptopodin at 4°C. Then, they were incubated with secondary antibodies and visualized with diaminobenzidine (DAB; brown color) and hematoxylin counterstaining under a microscope. The intensity of positive staining was determined according to the IOD/area value with Image-Pro Plus 6.0 image analysis software (Media Cybernetics, Silver Spring, MD). More than 10 fields for each section in each group were evaluated, and the mean value was used. All scoring was performed on blinded slides.

### 2.7. Immunofluorescence Staining

Immunofluorescence staining was performed using a rabbit anti-insulin polyclonal antibody to measure the activity of transplanted islets under the kidney capsule. The secondary antibody used for this analysis was changed to goat anti-rabbit IgG-FITC, and the nucleus was stained with Hoechst staining solution.

### 2.8. Transmission Electron Microscopy (TEM)

Small pieces of the renal cortex tissue block were fixed in 2.5% glutaraldehyde and washed with PBS (0.01 M). Then, they were postfixed with 1% osmium tetroxide, dehydrated with an acetone gradient, and embedded in Araldite M (Sigma Aldrich). Ultrathin sections were counterstained with uranyl acetate and lead citrate and examined with a transmission electron microscope (H-7700, Hitachi, Japan). The thickness of the GBM was measured with Image-Pro Plus 6.0 image analysis software.

### 2.9. Western Blotting Analysis

The proteins were extracted, and the total protein concentration was determined by Protein BCA Assay (Beyotime, Jiangsu, China). After blocking with 5% skim milk, the membranes were incubated overnight at 4°C with the primary antibodies. Then, they were incubated with goat anti-rabbit IgG (1 : 5000) conjugated to horseradish peroxidase (HRP) and visualized with an ECL Western Blotting Detection System (Amersham, Arlington Heights, IL, USA).

### 2.10. Statistical Analysis

All statistical data were analyzed using SPSS version 19.0 statistical software (SPSS, Chicago, IL USA), and the values are expressed as the mean ± standard deviation. Multiple comparisons between groups were performed by one-way ANOVA, and* post hoc* analyses were conducted using the least significant difference test. The differences between groups were considered significant at a *p* < 0.05.

## 3. Results

### 3.1. Evaluation of the Early DN Rat Model

**Figure 1:**
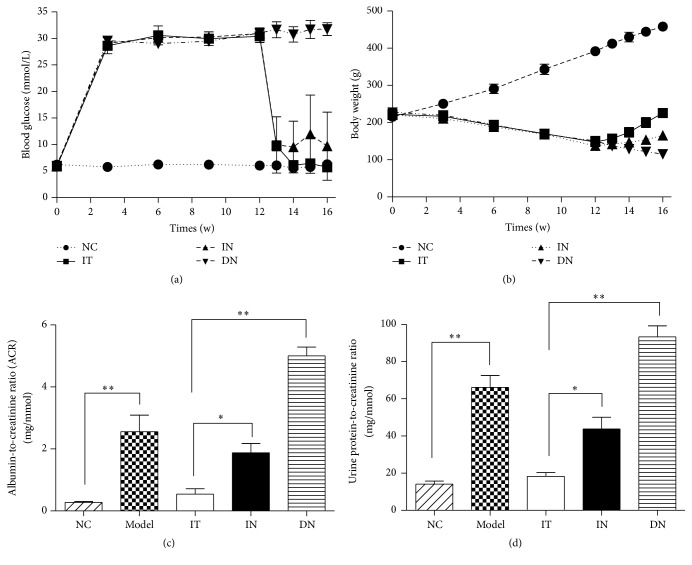
Blood glucose levels and body weights over 16 weeks and urinalysis results for each group. (a) Nonfasting blood glucose levels for each group. (b) Body weight changes over 16 weeks. (c) Random urine albumin-to-creatinine ratios (ACR) for each group, ^*∗*^ *p* = 0.002 and ^*∗∗*^ *p* < 0.001. (d) Urine protein-to-creatinine ratios for each group, ^*∗*^ *p* = 0.001 and ^*∗∗*^ *p* < 0.001. Model group: diabetic nephropathy model rats established at 12 weeks.

As shown in Figure 1, the urine microalbumin-to-creatinine ratio (ACR) and protein-to-creatinine ratio were determined to assess renal injury, and transmission electron microscopy (TEM) detection of the kidney tissue was performed to identify pathological changes. As local GBM thickening, podocyte depletion with fusion of foot processes, mesangial expansion, disordered endothelial cell arrangement, and glomerular filtration barrier structure abnormalities were evident, and the mean ACR (2.73 ± 0.58 mg/mmol) and urine protein-to-creatinine ratio (66.14 ± 7.25 mg/mmol) were significantly higher compared to those in the NC group (0.28 ± 0.06 mg/mmol and 14.37 ± 1.17 mg/mmol, resp. (Figures 1(c) and 1(d)), *p* < 0.05); the early DN rat model was considered to have been established successfully, as described previously [19]. Thus, this rat model was considered suitable for evaluating the advantageous effects of islet transplantation on early DN.

### 3.2. Islet Transplantation and Evaluation

**Figure 2:**
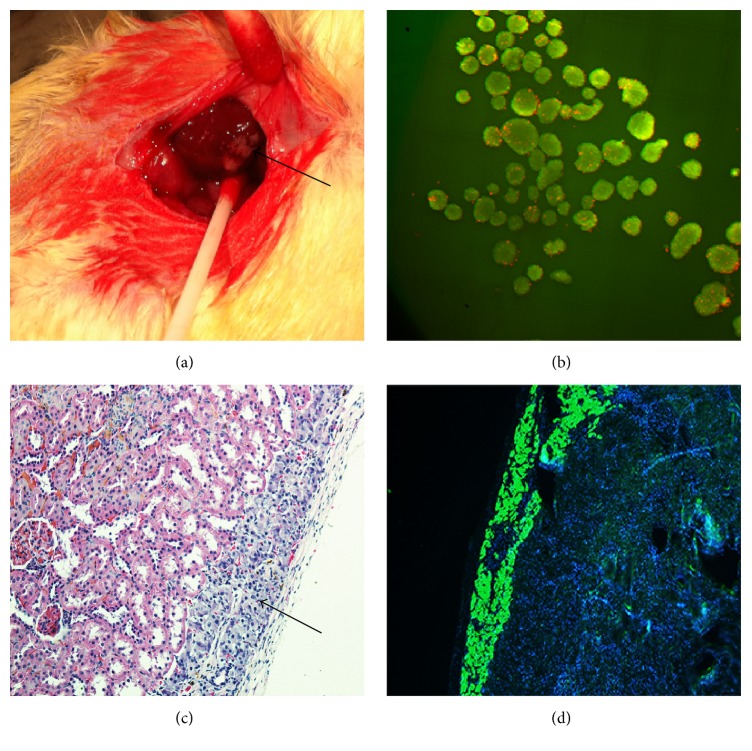
Islet transplantation under the kidney capsule and evaluation of islet activity. (a) Islets transplanted into the renal subcapsular space. (b) Activity evaluation of isolated islets (FDA-PI staining, ×100). (c) Transplanted islets under the kidney capsule (HE staining, ×200). (d) Insulin immunofluorescence staining (green, ×100).

Before transplantation, islet activity was evaluated by FDA-PI staining with an aliquot of islets, and the results revealed a high level of islet activity (>99%, Figure 2(b)). The transplanted islets were distributed and were visible under the capsule (Figure 2(a)). At four weeks after transplantation, immunohistochemical and immunofluorescence staining for insulin demonstrated high activity of the transplanted islets under the kidney capsule and indicated that they were still capable of normal insulin secretion (Figures 2(c) and 2(d)). Throughout this experiment, the surviving islets retained normal insulin secretion function and maintained the blood glucose level within the normal range; immunofluorescence staining verified these results.

### 3.3. Blood Glucose, Body Weight, and Urinary Parameters

At three days after transplantation, the blood glucose level in the IT group was significantly decreased and was maintained at a normal level (5.96 ± 1.81 mmol/L, Figure 1(a)). The body weights of the rats in the IT and IN groups gradually increased, whereas the growth curve for the IT group was significantly steeper than that for the IN group (Figure 1(b)). The glucose level in the rats in the DN group continued to remain high, along with a continuous decrease in body weight. As shown in Figure 1(c), after 4 weeks, the urinary ACR decreased significantly in the IT group (0.43 ± 0.11 mg/mmol) after islet transplantation. In contrast, it remained high in the IN group (1.64 ± 0.49 mg/mmol). Compared to the model group (2.73 ± 0.58 mg/mmol), the urinary ACR was higher (5.18 ± 1.36 mg/mmol) in the DN group. The urine protein-to-creatinine ratio also obviously differed between the IT and IN groups (Figure 1(d)). Compared with the IN (43.76 ± 6.30 mg/mmol) and DN groups (93.43 ± 8.51 mg/mmol), there was a significant decline in this ratio, which dropped close to the normal level (14.37 ± 1.17 mg/mmol), in the IT group (17.95 ± 2.20 mg/mmol).

### 3.4. TEM Examination of Glomerulus and Podocyte Density Evaluation

**Figure 3:**
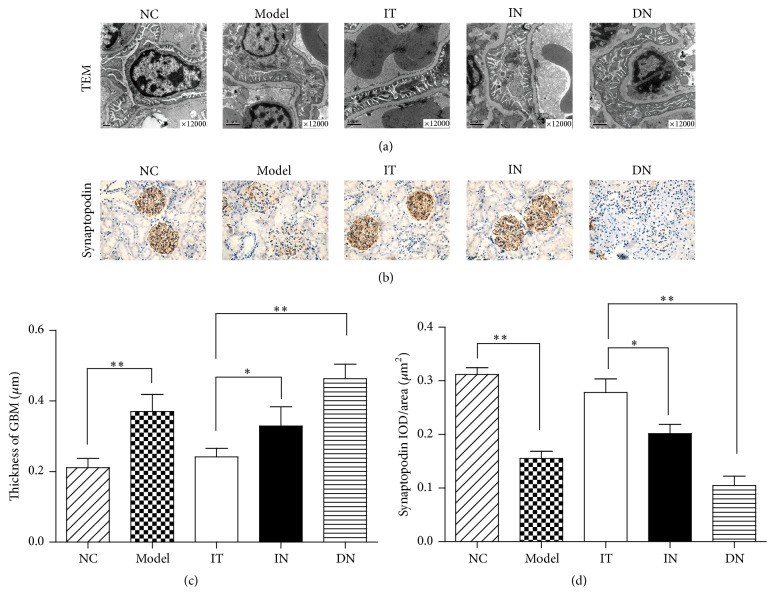
TEM detection of renal tissues and immunohistochemical staining for synaptopodin in podocytes. (a) TEM detection of podocytes and the glomerular filtration barrier. (b) Immunohistochemical staining for synaptopodin, which stains podocytes specifically, to measure the density of podocytes. (c) Measurement of the thickness of the GBM in each group, ^*∗*^ *p* = 0.012 and ^*∗∗*^ *p* < 0.001. (d) Comparison of the IOD/area of synaptopodin immunohistochemical staining for each group, ^*∗*^ *p* = 0.003 and ^*∗∗*^ *p* < 0.001. IOD/area, integrated optical density/area. The mean of IOD/area of positive staining was used to accurately measure the degree of positive expression in immunohistochemical staining. Model group: diabetic nephropathy model rats established at 12 weeks.

As shown in Figure 3(a), we found that the thickening of the GBM, disordered endothelial cell arrangement, and podocyte depletion with fusion of the foot processes were not obvious in the IT group compared with the NC group. In contrast, in the IN and DN groups, the range of podocyte depletion with fusion of the foot processes was more extensive than that in the IT group (Figure 3(a)). Additionally, as shown in Figure 3(c), the mean thickness of the GBM was measured, and the data demonstrated that the thickness in the IT group (0.24 ± 0.02 *μ*m) was significantly less than that in the IN group (0.33 ± 0.05 *μ*m, *p* = 0.001) or DN group (0.46 ± 0.04 *μ*m, *p* < 0.001). Synaptopodin stains podocytes specifically, and podocyte density can be measured by the intensity of synaptopodin staining. As shown in Figure 3(b), the intensity of synaptopodin staining was significantly decreased in the model and DN groups compared with the NC group. The strength of synaptopodin staining was obviously greater in the IT group than in the IN group, indicating a greater density of podocytes. This was also confirmed by the greater IOD/area value of synaptopodin staining in the IT group compared with the IN group (*p* = 0.003), as shown in Figure 3(d).

### 3.5. Assessment of Renal Fibrosis

**Figure 4:**
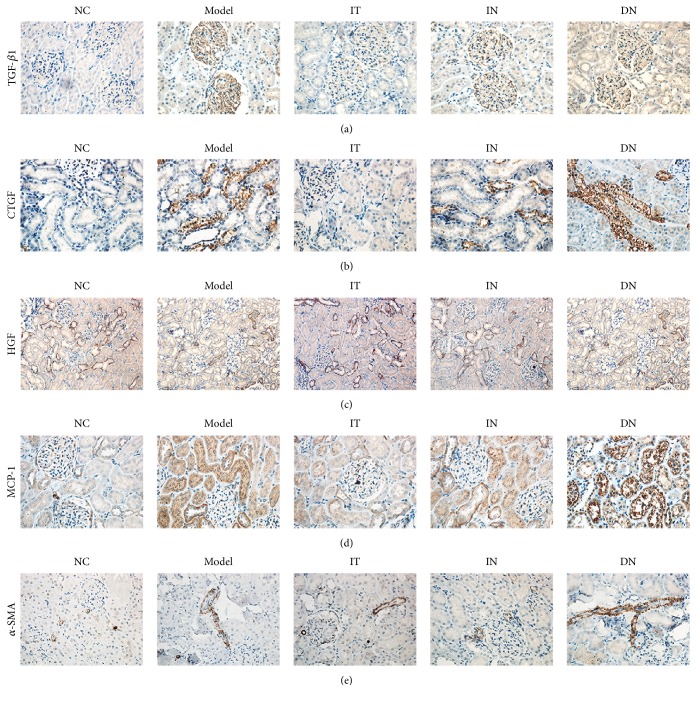
Immunohistochemical staining of renal fibrogenic and interstitial fibrosis factors in each group. (a) TGF-*β*1. (b) CTGF. (c) HGF. (d) MCP-1. (e) *α*-SMA. Model group: diabetic nephropathy model rats established at 12 weeks.

**Figure 5:**
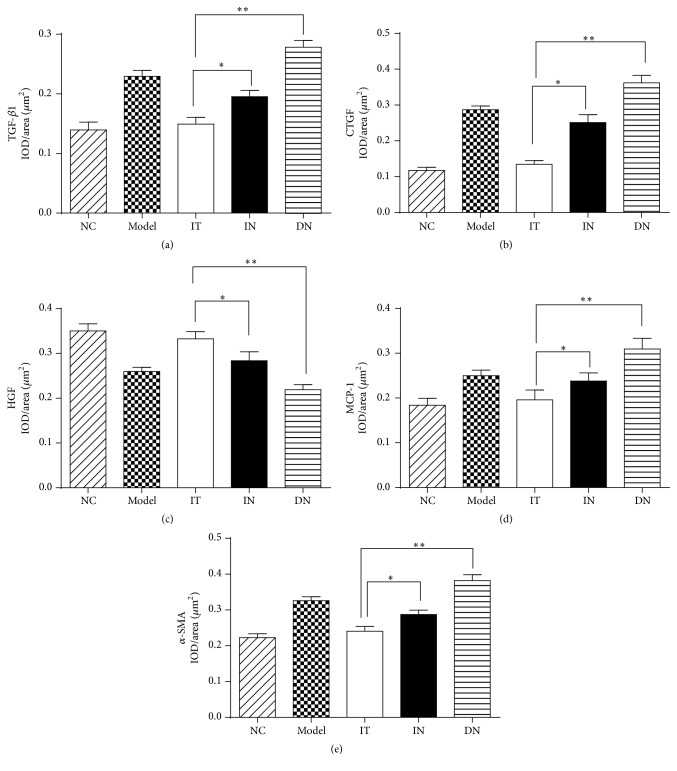
Measurement of IOD/area of immunohistochemical staining of renal fibrogenic and interstitial fibrosis factors. (a) Measurement of IOD/area of TGF-*β*1, ^*∗*^ *p* = 0.013, and ^*∗∗*^ *p* < 0.001. (b) Measurement of IOD/area of CTGF, ^*∗*^ *p* = 0.002 and ^*∗∗*^ *p* < 0.001. (c) Measurement of IOD/area of HGF, ^*∗*^ *p* = 0.001 and ^*∗∗*^ *p* < 0.001. (d) Measurement of IOD/area of MCP-1, ^*∗*^ *p* = 0.001 and ^*∗∗*^ *p* < 0.001. (e) Measurement of IOD/area of *α*-SMA, ^*∗*^ *p* = 0.018 and ^*∗∗*^ *p* < 0.001. IOD/area, integrated optical density/area. The mean of IOD/area of positive staining was used to accurately measure the degree of positive expression in immunohistochemical staining. Model group: diabetic nephropathy model rats established at 12 weeks.

**Figure 6:**
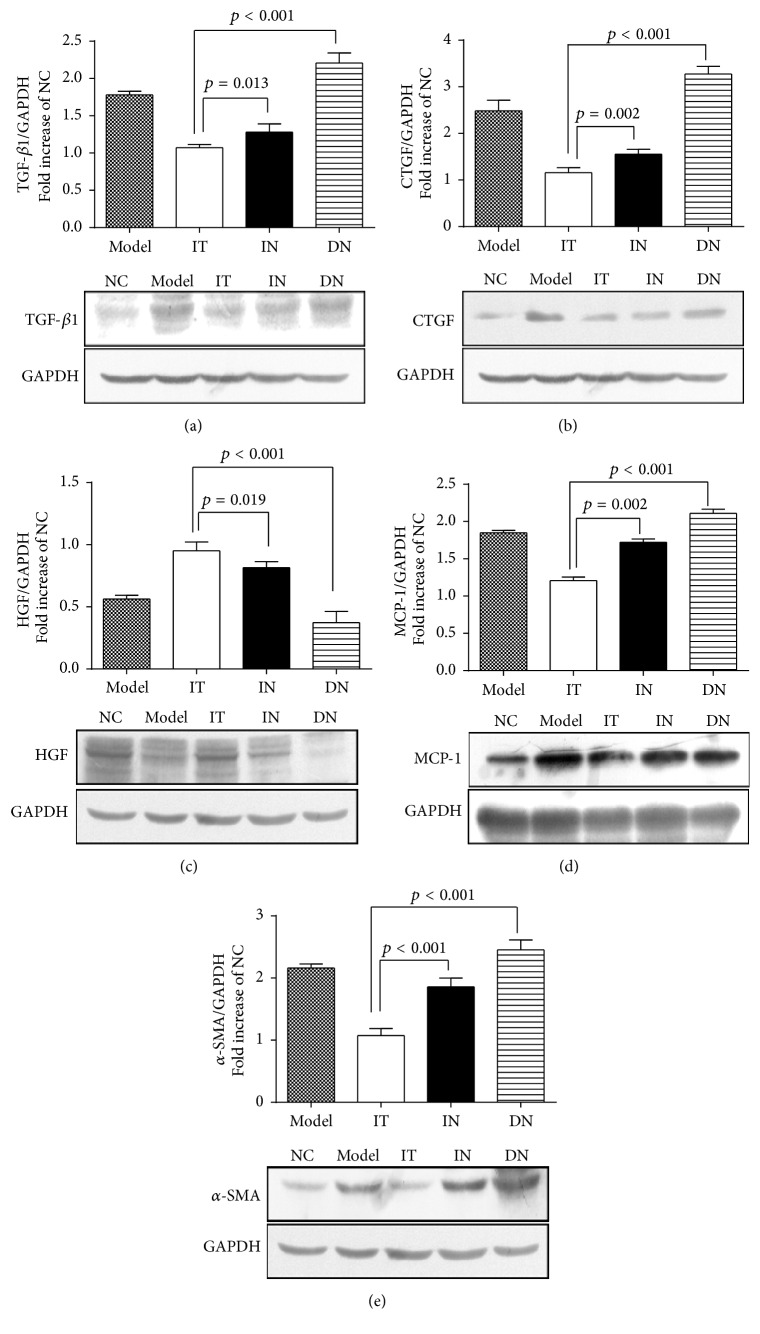
Western blotting analysis of fibrogenic and interstitial fibrosis factors in renal tissues. Western blotting analysis was performed to assess the protein expression of fibrogenic and interstitial fibrosis factors in the kidneys in each group. (a) TGF-*β*1. (b) CTGF. (c) HGF. (d) MCP-1. (e) *α*-SMA. Model group: diabetic nephropathy model rats established at 12 weeks.

To confirm and compare the ameliorative effects of islet transplantation and insulin therapy on renal fibrosis in early DN, immunohistochemical staining was performed to determine the expression of fibrosis-related factors. As shown in Figures 4 and 5, decreased expression of TGF-*β*1, CTGF, MCP-1, and *α*-SMA and increased expression of HGF were detected in the IT group compared with the IN group (*p* < 0.05 for each). Additionally, the brown staining of the granules was significantly decreased in the IT group compared with the DN group, and the IT group also had a lower mean IOD/area value for the related renal fibrosis factors (*p* < 0.001). Western blotting analysis further demonstrated that the protein expression of each factor was obviously lower in the IT group compared with the IN and DN groups (Figure 6); these results coincide with the immunohistochemical staining results.

## 4. Discussion

Over the past few decades, islet transplantation has emerged as an alternative therapeutic method for the use of conventional antidiabetic drugs and insulin for the treatment of diabetic patients [20]. Several studies have demonstrated that pancreas or islet transplantation is an effective treatment for diabetic patients with complications. In recent years, remarkable results have also been observed in the clinical application of islet transplantation in humans. Researchers have demonstrated that the kidney graft survival rates for uremic patients with type 1 diabetes mellitus can improve significantly after successful islet transplantation [21]. These findings further demonstrated that islet transplantation is an effective treatment for diabetic nephropathy. Our study was designed to investigate the efficacy of islet transplantation for reversing early DN and to establish whether this treatment is superior to insulin therapy for treating diabetic complications in model rats. The early DN model was successfully established as described previously [19]. Throughout the study, the transplanted islets were able to maintain the blood glucose level within the normal range without the use of immunosuppressants in SD rats with diabetic nephropathy. These findings are in agreement with those of Ar'Rajab and colleagues [13]. The results of our study demonstrated that all of the measured clinical signs of diabetes mellitus were abolished after islet transplantation and that renal injury was significantly improved; these signs included a decrease in the blood glucose level, attenuation of proteinuria and albuminuria, and normalization of the glomerular filtration barrier. Further, histopathological staining revealed that renal fibrosis was obviously alleviated after islet transplantation. In contrast, with insulin treatment, the blood glucose level, ACR, and protein-to-creatinine ratio were not well controlled, and the expression of related renal fibrosis factors was obviously higher than that observed following islet transplantation in this study. All of these data demonstrated that early DN could be reversed after islet transplantation, and the ameliorative effects achieved with transplantation were significantly better than those obtained with insulin therapy for the treatment of diabetic complications. Furthermore, TEM was also used in this study to detect microscopic changes of the glomerular basement membrane (GBM) and podocytes in the glomerulus. Wide application of this technology would allow for more accurate analysis of kidney diseases in the clinical setting. Changes of microscopic structures (podocytes and GBM) are considered to play an important role during the progression of renal disease.

Podocytes are highly differentiated epithelial cells in the glomerulus that attach to the GBM to maintain efficient glomerular filtration. Their interdigitating foot processes are bridged by a slit diaphragm to control the patency through the transcytotic clearance mechanisms [22]. Recent research has indicated that podocyte damage plays a crucial role in progressive DN and that it can directly result in progressive renal hypofunction [22, 23]. In the early stage of diabetes, podocyte dysfunction is manifested histologically by the broadening of foot processes and functionally by various physiological alterations. Thickening of the GBM is also an important characteristic of the progression of DN. Injury to the GBM or podocytes could cause barrier damage and protein loss, which might explain why progressive proteinuria was the typical manifestation of early DN [24]. In our study, islet transplantation significantly ameliorated the thickening of the GBM and the podocyte depletion with fusion of the foot processes. These results coincided with the improvement in renal function and attenuation of kidney damage. Growing evidence indicates that the signaling abnormalities in podocytes in DN mainly involve the TGF-*β* family [25]. The blockage of inflammation-induced injury or intervention of inflammatory mechanisms in podocytes could ameliorate renal fibrosis in early DN [26].

Renal fibrosis is one of the typical features of early DN, and the degree of fibrosis is strongly associated with the progression of DN [27]. Progressive accumulation of myofibroblasts in the glomerulus and tubules is critical for the development of DN; therefore, early prevention or intervention of renal fibrosis is extremely important. Studies have also indicated that the actions of fibrotic factors and cytokines, such as TGF-*β*1, HGF, CTGF, *α*-SMA, and MCP-1, play significant roles in the progression of renal fibrosis in early DN. Compared with the untreated DN rats, the expression of HGF was obviously elevated, accompanied by a significant decrease in the expression of other factors, after islet transplantation. TGF-*β*1 is regarded as the central fibrogenic factor in the pathogenesis of progressive renal fibrosis in DN. Its overexpression stimulates renal fibrosis by promoting podocyte and tubular epithelial and glomerular endothelial cell apoptosis, activating interstitial fibroblasts, and inducing the activation of *α*-SMA expression and mesangial cells to produce large amounts of ECM components [28]. HGF might inhibit TGF-*β*1 expression and prevent the progression of renal fibrosis in various animal models [29]. Components of the diabetic milieu also could promote the recruitment of macrophages by stimulating the expression of MCP-1 in the kidneys in response to renal injury [30]. The results of our study showed that renal fibrosis was improved significantly after islet transplantation compared with that observed after insulin therapy.

Because it is associated with a lower risk of surgery and fewer side effects than full-pancreas transplantation, pancreatic islet transplantation had been widely investigated as a promising strategy for treating diabetes mellitus and/or its complications. It is well known that sustained hyperglycemia is one of the major causes of DN. Regular oral insulin administration or insulin injection cannot always maintain plasma glucose within the normal range, resulting in fluctuations in the blood glucose level [31, 32]. The kidneys remain in a suboptimal state when hyperglycemia cannot be eliminated. However, because islet transplantation is capable of physiologic regulation* in vivo*, it can control the secretion of insulin automatically to maintain the blood glucose level within the normal range. Hypoglycemia is considered one of the most dangerous complications occurring in clinically diabetic patients, and intensive insulin therapy protocols have been widely used in the clinical intensive care of diabetes mellitus; notably, the incidence of hypoglycemia is markedly increased if the blood glucose level is not constantly monitored [33]. Nevertheless, because of the self-regulation of physiological systems, excessive insulin secretion does not occur after islet transplantation. During this study, although a rat's blood glucose level was not fully maintained within the normal range after transplantation, the renal damage and fibrosis of DN of this rat were still alleviated as observed in the late examination. These findings could indicate that transplantation of a small amount of transplanted islets also had ameliorative effects on DN, but further study is required for clarification.

Relevant signaling pathways about the ameliorative effects of islet transplantation have not been illuminated very clearly, and these topics also will be the main focuses of our next experiments. Recent studies have demonstrated that abnormalities in signaling pathways could also contribute to the pathologic signs of DN. An increasing number of studies have attempted to elucidate the molecular mechanisms of DN to facilitate the development of preventative strategies and effective therapies [34]. Fiorina and his colleagues have found the induction of immune-related protein B7-1 is associated with the progression of proteinuria in human and animal glomerular diseases. The expression of B7-1 in podocytes is upregulated in high glucose conditions, which can induce podocyte morphologic abnormalities and promote cell death. Targeting B7-1 has been suggested as a promising effective method on preventing cure diabetic nephropathy in their studies [35]. These new pathogenic mechanisms have been clarified by manipulation of gene expression in animal models, and the use of such models might contribute to the understanding and clinical therapy of DN. Due to the limitations of the present study, many important issues remain to be addressed, particularly the survival of the transplanted islets and the minimum equivalent required to treat diabetes mellitus. Meanwhile, research has indicated that C-peptide might play an important role in the control of blood glucose in diabetic patients. Johansson et al. [36] have found that the combined administration of insulin, proinsulin, and C-peptide has greater efficacy for the treatment of diabetes than insulin injection alone. Whether islet transplantation can regulate the secretion of C-peptide is also a vital issue [37]. The encouraging studies and results of the present study could provide a basis for the clinical application of islet transplantation.

In conclusion, our results demonstrated that islet transplantation could reverse various symptoms of early DN in a rat model, and the ameliorative effects on kidney injury and renal fibrosis were obviously better with islet transplantation than with insulin therapy. The recovery of impaired podocyte structure and function is the key to the treatment of diabetic nephropathy. In this study, islet transplantation under the kidney capsule significantly ameliorated the impaired renal function and microscopic damage in the early diabetic nephropathy rats. These findings may provide new possibilities to treat or prevent early DN and other complications in patients with diabetes mellitus in the future.

## References

[1] Yokoyama H., Deckert T. (1996). Central role of TGF-*β* in the pathogenesis of diabetic nephropathy and macrovascular complications: a hypothesis. *Diabetic Medicine*.

[2] Svensson M., Sundkvist G., Arnqvist H. J. (2003). Signs of nephropathy may occur early in young adults with diabetes despite modern diabetes management: results from the nationwide population-based Diabetes Incidence Study in Sweden (DISS). *Diabetes Care*.

[3] Liu Z. H. (2013). Nephrology in China. *Nature Reviews Nephrology*.

[4] Kim S. S., Kim J. H., Kim I. J. (2016). Current challenges in diabetic nephropathy: early diagnosis and ways to improve outcomes. *Endocrinology and Metabolism*.

[5] van der Sande N. G., Dorresteijn J. A., Visseren F. L. (2016). Individualized prediction of the effect of angiotensin receptor blockade on renal and cardiovascular outcomes in patients with diabetic nephropathy. *Diabetes, Obesity and Metabolism*.

[6] Li F.-F., Fu L.-Y., Zhang W.-L. (2016). Blood glucose fluctuations in type 2 diabetes patients treated with multiple daily injections. *Journal of Diabetes Research*.

[7] Saidi R. F. (2012). Current status of pancreas and islet cell transplantation. *International Journal of Organ Transplantation Medicine*.

[8] Lundberg J., Stone-Elander S., Zhang X.-M., Korsgren O., Jonsson S., Holmin S. (2014). Endovascular method for transplantation of insulin-producing cells to the pancreas parenchyma in swine. *American Journal of Transplantation*.

[9] Fioretto P., Mauer M. (2011). Effects of pancreas transplantation on the prevention and reversal of diabetic nephropathy. *Contributions to Nephrology*.

[10] Fiorina P., Perseghin G., De Cobelli F. (2007). Altered kidney graft high-energy phosphate metabolism in kidney-transplanted end-stage renal disease type 1 diabetic patients: a cross-sectional analysis of the effect of kidney alone and kidney-pancreas transplantation. *Diabetes Care*.

[11] Fiorina P., Venturini M., Folli F. (2005). Natural history of kidney graft survival, hypertrophy, and vascular function in end-stage renal disease type 1 diabetic kidney-transplanted patients: beneficial impact of pancreas and successful islet cotransplantation. *Diabetes Care*.

[12] Brosius F. C., Khoury C. C., Buller C. L., Chen S. (2010). Abnormalities in signaling pathways in diabetic nephropathy. *Expert Review of Endocrinology and Metabolism*.

[13] Ar'Rajab A., Ahren B., Alumets J., Logdberg L., Bengmark S. (1990). Islet transplantation to the renal subcapsular space improves late complications in streptozotocin-diabetic rats. *European Surgical Research*.

[14] Araiza-Saldaña C. I., Pedraza-Priego E. F., Torres-Lõpez J. E. (2015). Fosinopril prevents the development of tactile allodynia in a streptozotocin-induced diabetic rat model. *Drug Development Research*.

[15] Zmuda E. J., Powell C. A., Hai T. (2011). A method for murine islet isolation and subcapsular kidney transplantation. *Journal of Visualized Experiments*.

[16] Lembert N., Wesche J., Petersen P., Doser M., Becker H. D., Ammon H. P. T. (2003). Areal density measurement is a convenient method for the determination of porcine islet equivalents without counting and sizing individual islets. *Cell Transplantation*.

[17] Napoli R., Davalli A. M., Hirshman M. F., Weitgasser R., Weir G. C., Horton E. S. (1996). Islet transplantation under the kidney capsule fully corrects the impaired skeletal muscle glucose transport system of streptozocin diabetic rats. *The Journal of Clinical Investigation*.

[18] Pichaiwong W., Hudkins K. L., Wietecha T. (2013). Reversibility of structural and functional damage in a model of advanced diabetic nephropathy. *Journal of the American Society of Nephrology*.

[19] Tesch G. H., Allen T. J. (2007). Rodent models of streptozotocin-induced diabetic nephropathy. *Nephrology*.

[20] Pepper A. R., Pawlick R., Gala-Lopez B. (2015). Diabetes is reversed in a murine model by marginal mass syngeneic islet transplantation using a subcutaneous cell pouch device. *Transplantation*.

[21] Fiorina P., Folli F., Zerbini G. (2003). Islet transplantation is associated with improvement of renal function among uremic patients with type I diabetes mellitus and kidney transplants. *Journal of the American Society of Nephrology*.

[22] Lu M.-K., Gong X.-G., Guan K.-L. (2011). mTOR in podocyte function: is rapamycin good for diabetic nephropathy?. *Cell Cycle*.

[23] Niranjan T., Bielesz B., Gruenwald A. (2008). The Notch pathway in podocytes plays a role in the development of glomerular disease. *Nature Medicine*.

[24] Alsaad K. O., Herzenberg A. M. (2007). Distinguishing diabetic nephropathy from other causes of glomerulosclerosis: an update. *The Journal of Clinical Pathology*.

[25] Iglesias-de la Cruz M. C., Ziyadeh F. N., Isono M. (2002). Effects of high glucose and TGF-*β*1 on the expression of collagen IV and vascular endothelial growth factor in mouse podocytes. *Kidney International*.

[26] Awad A. S., Huang L., Ye H. (2006). Adenosine A2A receptor activation attenuates inflammation and injury in diabetic nephropathy. *American Journal of Physiology-Renal Physiology*.

[27] McClelland A. D., Herman-Edelstein M., Komers R. (2015). miR-21 promotes renal fibrosis in diabetic nephropathy by targeting PTEN and SMAD7. *Clinical Science*.

[28] Schnaper H. W., Hayashida T., Hubchak S. C., Poncelet A.-C. (2003). TGF-*β* signal transduction and mesangial cell fibrogenesis. *American Journal of Physiology—Renal Physiology*.

[29] Bessho K., Mizuno S., Matsumoto K., Nakamura T. (2003). Counteractive effects of HGF on PDGF-induced mesangial cell proliferation in a rat model of glomerulonephritis. *American Journal of Physiology—Renal Physiology*.

[30] Chow F. Y., Nikolic-Paterson D. J., Ozols E., Atkins R. C., Rollin B. J., Tesch G. H. (2006). Monocyte chemoattractant protein-1 promotes the development of diabetic renal injury in streptozotocin-treated mice. *Kidney International*.

[31] Kim K.-W. (2004). Islet transplantation: a realistic alternative for the treatment of insulin deficient diabetes mellitus. *Diabetes Research and Clinical Practice*.

[32] Tiemessen C. A. M., Hoedemaekers C. W. E., Van Iersel F. M. (2011). Intensive insulin therapy increases the risk of hypoglycemia in neurocritical care patients. *Journal of Neurosurgical Anesthesiology*.

[33] Czech M., Rdzanek E., Pawȩska J., Adamowicz-Sidor O., Niewada M., Jakubczyk M. (2015). Drug-related risk of severe hypoglycaemia in observational studies: a systematic review and meta-analysis. *BMC Endocrine Disorders*.

[34] Qian Y., Feldman E., Pennathur S., Kretzler M., Brosius F. C. (2008). From fibrosis to sclerosis: mechanisms of glomerulosclerosis in diabetic nephropathy. *Diabetes*.

[35] Fiorina P., Vergani A., Bassi R. (2014). Role of podocyte B7-1 in diabetic nephropathy. *Journal of the American Society of Nephrology*.

[36] Johansson B.-L., Borg K., Fernqvist-Forbes E., Kernell A., Odergren T., Wahren J. (2000). Beneficial effects of C-peptide on incipient nephropathy and neuropathy in patients with Type 1 diabetes mellitus. *Diabetic Medicine*.

[37] Blau J. E., Abegg M. R., Flegel W. A., Zhao X., Harlan D. M., Rother K. I. (2015). Long-term immunosuppression after solitary islet transplantation is associated with preserved C-peptide secretion for more than a decade. *American Journal of Transplantation*.

